# Overexpression of Extracellular Superoxide Dismutase Protects against Brain Injury Induced by Chronic Hypoxia

**DOI:** 10.1371/journal.pone.0108168

**Published:** 2014-09-30

**Authors:** Nahla Zaghloul, Hardik Patel, Champa Codipilly, Philippe Marambaud, Stephen Dewey, Stephen Frattini, Patricio T. Huerta, Mansoor Nasim, Edmund J. Miller, Mohamed Ahmed

**Affiliations:** 1 Division of Neonatal-Perinatal Medicine, The Ohio State University and Nationwide Children's Hospital, Columbus, Ohio, United States of America; 2 Laboratory of Memory Disorders, Feinstein Institute for Medical Research, Manhasset, New York, United States of America; 3 Neuroimaging Department, Feinstein Institute for Medical Research, Manhasset, New York, United States of America; 4 Laboratory of Immune & Neural Networks, Feinstein Institute for Medical Research, Manhasset, New York, United States of America; 5 Department of Molecular Medicine, Hofstra North Shore LIJ School of Medicine, New York, United States of America; 6 Department of Pathology, NSL-IJ, Manhasset, New York, United States of America; 7 Division of Neonatal-Perinatal Medicine, Cohen Children's Medical Center of New York and Lilling Family Research laboratory, Feinstein Institute for Medical Research, Manhasset, New York, United States of America; University of Pittsburgh, United States of America

## Abstract

Extracellular superoxide dismutase (EC-SOD) is an isoform of SOD normally found both intra- and extra-cellularly and accounting for most SOD activity in blood vessels. Here we explored the role of EC-SOD in protecting against brain damage induced by chronic hypoxia. EC-SOD Transgenic mice, were exposed to hypoxia (FiO2.1%) for 10 days (H-KI) and compared to transgenic animals housed in room air (RA-KI), wild type animals exposed to hypoxia (H-WT or wild type mice housed in room air (RA-WT). Overall brain metabolism evaluated by positron emission tomography (PET) showed that H-WT mice had significantly higher uptake of ^18^FDG in the brain particularly the hippocampus, hypothalamus, and cerebellum. H-KI mice had comparable uptake to the RA-KI and RA-WT groups. To investigate the functional state of the hippocampus, electrophysiological techniques in *ex vivo* hippocampal slices were performed and showed that H-KI had normal synaptic plasticity, whereas H-WT were severely affected. Markers of oxidative stress, GFAP, IBA1, MIF, and pAMPK showed similar values in the H-KI and RA-WT groups, but were significantly increased in the H-WT group. Caspase-3 assay and histopathological studies showed significant apoptosis/cell damage in the H-WT group, but no significant difference in the H-KI group compared to the RA groups. The data suggest that EC-SOD has potential prophylactic and therapeutic roles in diseases with compromised brain oxygenation.

## Introduction

Hypoxia plays a crucial role in acute and chronic CNS pathologies. Exposure to hypoxia results in a significant increase in reactive oxygen species (ROS), including superoxide, which is produced mainly in the mitochondria [1]–[4]. ROS leads to impaired neurogenesis, hippocampal atrophy, altered transcription factor regulation, and protein expression [5]–[6].

Excess ROS, particularly superoxide, can oxidize nitric oxide (NO) to reactive nitrogen species (RNS) including peroxynitrite [7]–[8]. This process leads to decreased NO bioavailability, accumulation of toxic products including NO2 [9]–[10]. Both ROS and RNS oxidize macromolecules (DNA, proteins, and lipids), culminating in CNS neurodegeneration [11]. Oxidative stress also activates glial-mediated inflammation [12]. HIF-1 alpha rapidly accumulates during the onset of hypoxia, remaining at this level for 14 days before gradually declining to normal by 21 days despite continuous hypoxia [13]–[14]. A pathophysiological role for HIF-1alpha has been established for hypoxic ischemic diseases [15].

Neurons are particularly susceptible to ROS/RNS injury [16], but may adapt to hypoxia by activating neuroprotective signaling cascades e.g. MAPK, ERK1/2, and protein kinase-B [17]–[18] increasing glycolytic energy metabolism and free-radical defenses [19], down-regulating oxidative-stress genes, and up-regulating antioxidant genes [20].

Overexpression of mitochondrial SOD2 inhibits post-ischemic mitogen-activated protein kinase and decreases DNA fragmentation following ischemia/reperfusion [21]–[25]. The outcome from middle cerebral artery occlusion is worse in SOD2 deficient animals [26]. A neuroprotective role for SOD mimetics has been demonstrated in an *ex-vivo* brain model [27].

EC-SOD is expressed in the brain at a lower level than other SODs [28], but provides defense against ROS produced by membrane-bound NAD(P)H oxidase [29]. EC-SOD is predominantly localized in neurons of hippocampus, lateral nucleus of the thalamus and hypothalamus. Both EC-SOD and neuronal NO synthase are similarly distributed in the striatum and cortex [30]. EC-SOD is the only extracellular SOD isoform and the major SOD activity in blood vessels, which leads to increase NO bioavailability [31].

Mice, engineered to overexpress EC-SOD, have increased tolerance to both focal and global cerebral ischemia [32]–[34], while EC-SOD knock-outs exhibit enhanced damage [35]. These data implicate an important role for EC-SOD ischemia/reperfusion pathologies, and suggest a therapeutic role for SOD mimetics.

Previously, we showed that EC-SOD offers significant protection against oxidative stress-induced lung injury [36]–[37] and brain injury induced by hyperoxia [38]. In this study, we hypothesized that EC-SOD overexpression offers protection to the brain exposed to chronic hypoxia. This could be of importance to many diseases with compromised brain oxygenation.

## Material and Methods

All experiments involving animals were reviewed and approved by the Institutional Animal Care and Use Committee of the Feinstein Institute for Medical Research, and performed in accordance with the guidelines set by the Institute for Laboratory Animal Research. Adult C57BL6 mice, (8–10 week old), either wild type (WT) or transgenic animals (KI) generated by microinjecting nuclei of fertilized oocytes from (C57Bl/6#C3H), with the cDNA of human EC-SOD containing a Beta-actin promoter [29], were housed in a pathogen-free environment, under standard light and dark cycles, with free access to food and water. An animal hypoxia chamber system (BioSpherix, Lacona, NY) was used for the *in vivo* studies. With this system, a constant 10% normobaric hypoxia was achieved for up to 10 days in our study. Animals were divided into four groups (10/group) and housed for 10 days as follows: Group A: WT adult mice housed in room air (RA-WT). Group B: KI adult mice housed in room air (RA-KI). Group C: WT adult mice housed in hypoxia (H-WT). Group D: KI adult mice housed in hypoxia (H-KI). After 10 days, the animals were assessed using PET, and were then euthanized and brain tissues harvested.

### Functional studies

#### 1- PET scanning

Brains were scanned after exposure to hypoxia for 10 days and compared to matched room air controls. Scanning was performed using ^18^FDG, which was injected intraperitoneally at a dose of 400 Micro Curie per mouse. After 45 min., the mice were assessed at the PET scan center located within the Feinstein Institute for Medical Research.

#### 2- Hippocampal Electrophysiology

Mice were anesthetized with isoflurane in a closed container, then immediately decapitated. The brain was quickly extracted into ice-cold (<2C) artificial cerebral spinal fluid (ACSF) that contained: NaCl (126 mM), NaHCO_3_ (26 mM), glucose (10 mM), KCl (2.5 mM), CaCl_2_ (2.4 mM), MgCl_2_ (1.3 mM), and NaH_2_PO_4_ (1.2 mM), and was continuously gassed with carbogen (95% O_2_, 5% CO_2_). Kynurenic acid (1 mM), which is a non-specific blocker of excitatory amino acid receptors, was added to the ACSF solution during the dissection and slicing procedures. The brain was bisected and both hemispheres were mounted onto a block with ethyl cyanoacrylate glue. Transverse hippocampal slices (400 mm thick) were prepared using a Leica VT1200 vibratome. Slices were incubated in ACSF gassed with carbogenfor 35 min at 35C followed by 120 min at 24C. One slice at a time was transferred to a recording chamber, continuously perfused with ACSF gassed with carbogen at 30C, for electrophysiological studies. Field excitatory postsynaptic potentials (fEPSP) were recorded with borosilicate glass electrodes (2–3 M ohmns tip resistance) placed in the stratum radiatum, of the CA1, at the midpoint between two bipolar stimulating electrodes (Frederick Haer & Co, Bowdoinham, ME) that activated Schaeffer collateral/commissural axons. This setup allowed for the recording of two independent pathways (test and control) in the same slice. The initial slope of the fEPSP was used as a measure of the postsynaptic response. fEPSP responses were amplified (AM Systems 1800), digitized at 10 kHz, and analyzed online with custom software (written with AxoBasic, Axon Instruments, Union City, CA). For obtaining input-output (I-O) functions, the stimulation was reduced to a value at which no fEPSP was evoked. The stimulation was then increased incrementally to evoke larger fEPSPs. This was done until the appearance of a population spike, generated by CA1 pyramidal cells, which defined the final point of the I-O function. For plasticity experiments, a stable baseline was obtained for at least 15 min. The baseline intensity was set to obtain a fEPSP slope that was half-maximal, as determined by I-O curves. Long-term potentiation (LTP) was induced by high-frequency stimulation (HFS), which consisted of either tetanus (100 Hz for 1 sec) or theta burst stimulation (TBS, 10 trains of 4 pulses at 100 Hz, with 200 ms between trains). We calculated LTP from 30 responses at 40–45 min post-HFS. For all LTP experiments, picrotoxin (100 microM) was added to block GABA_A_ receptors [39].

### Molecular studies

#### 1- Superoxide dismutase (SOD) assays

After PET scanning, all animals were euthanized. Brains were isolated and immediately frozen. SOD activity was assessed in brain homogenates using an Oxiselect superoxide dismutase activity assay kit (Cell Biolabs, Dan Diego, CA). This assay utilizes a xanthine/xanthine oxidase system to generate superoxide anions, which reduce a chromagen to a water soluble formazan dye. The activity of SOD in the brain tissue homogenate was determined as the inhibition of chromagen reduction. Quantitative assays of SOD1, SOD2,and hSOD3 were done by western blot and compared to B-Actin protein concentration.

#### 2- Glutathione assay

For the determination of the reduced glutathione (GSH) and oxidized glutathione (GSSG) ratio, the brain was homogenized and the homogenates were treated with a mixture of metaphosphoric acid, EDTA, and NaCl. After centrifugation, aliquots were taken for neutralization with disodium hydrogen phosphate followed by addition of DTNB. Reduced and oxidized glutathione were measured in brain tissue homogenates by reaction with DTNB (5,5′-dithiobis-2-nitrobenzoic acid) using a Glutathione Assay Kit (Calbiochem, Gibbsontown, NJ), following the manufacturer's instructions. Reduced glutathione (GSH) was determined after reaction with DTNB in a spectrophotometer at 412 nm. For the determination of oxidized glutathione (GSSG), the autoxidation of GSH was stopped by addition of N-ethylmaleimide. After addition of sodium hydroxide, GSSG was modified using o-phthalaldehyde. GSSG was determined at a spectrofluorometer (excitation: 350 nm, emission: 420 nm) using GSSG standards for quantification [40].

#### 3- Western Blotting

Frozen brain tissues were crushed and homogenized, and protein extraction was carried out using a Total Protein Extraction Kit (BioChain Institute, Inc. Hayward, CA). Protein concentration was estimated using the Modified Lowry Protein Assay (Thermo Fisher Scientific, Rockford, IL, USA). Samples were prepared for SDS-PAGE in Laemmli Sample Buffer (Bio-rad, Hercules, CA, USA). Standard SDS-PAGE techniques were followed as previously described [38]. Running buffer and Transfer buffer were purchased from Bio-Rad (Hercules, CA, USA). Briefly electrophoresis was performed using a Mini Format 1-D Electrophoresis Systems (Bio-Rad, Hercules, CA, USA) on 10–12% ready tris-HCl gels (Bio-rad, Hercules, CA, USA). After electrophoresis, proteins were transferred to a PVDF membrane using a Wet/Tank Blotting System (Bio-rad, Hercules, CA, USA). Membranes were briefly washed and immediately incubated with respective primary antibody in 5% BSA with PBST overnight (primary antibody was diluted according to the manufacturer's recommendation). The next day, after washing, the membranes were incubated with HRP-conjugated secondary antibodies for 40–60 min (diluted according to the manufacturer's recommendation). After incubation with secondary antibody, the membranes were washed and then processed using Amersham ECL detection systems (GE healthcare, Piscataway, NJ USA). The membranes were then immediately exposed to 8×10 Fuji X-Ray Film. Developed films were quantified using Quantity One 1-D Analysis Software on a GS-800 Calibrated Densitometer. The density of each band was evaluated and presented as a ratio in comparison to Actin band density. The following primary antibodies were used to detect the following markers: GFAP (Cell Signaling Technology, Danvers, MA, USA), Iba-1 (Wako Chemicals USA, Richmond, VA, USA), MIF (Abcam, Cambridge, MA, USA), pAMPK (Beauchamp et al., 2004), (Cell Signaling Technology, Danver, MA), pAMPK (Cell Signaling Technology, Danver, MA, USA), and anti-Actin protein (as an internal control) (Gene Script, Olathe, KS, USA). Horseradish Peroxidase (HRP)-Conjugated Goat Anti-Rabbit IgG conjugate was used for detection of rabbit primary antibodies (Bio-Rad, Hercules, CA, USA). Goat anti-mouse HRP conjugates were used for detection of mouse primary antibodies (Southern Biotech, Birmingham, AL, USA).

### Structural studies

#### 1- Caspase 3 activity assay

Caspase 3 activity was measured using the Caspase-3 Colorimetric Assay (R&D Systems, Minneapolis, MN, USA). The assay was carried out following the manufacturer's instructions and described previously [41]. Brain tissue samples were homogenized in buffer containing 10 mM HEPES (pH 7.4), 42 mM KCl, 5 mM MgCl2, 1 mM dithiothreitol, 1% Triton X-100, 0.5% CHAPS, 1 mM phenyl methyl sulfonyl fluoride, and 1 micro gm/mL leupeptin, and centrifuged at 12,000 *g* for 10 minutes at 4°C. A 10-µL aliquot of the lysate was incubated in a flat bottom 96 well plate, 50 microL tissue lysates were incubated with 50 microL 2x reaction buffer followed by 5 microL of caspase-3 colorimetric substrate (DEVD-pNA), and incubated at 37C for 2 hrs. The fluorescence was measured at room temperature at the excitation wavelength of 360 nm, and emission was measured at 460 nm with the use of a multiplate fluorescence reader (Biotek Instruments). Protein concentration was measured with a Pierce kit (Pierce Biotechnology, Rockford, IL, USA). Ac-AMC was used to obtain a standard curve. Enzyme activity was calculated as picomoles per minute per mg of protein.

#### 2- Histopathological studies

Brain tissue was fixed in 10% neutral buffered formalin for 24 hours, processed, embedded in paraffin wax, and subsequently cut into 4 micron thick sections. Following de-paraffinization, hematoxylin and eosin (H&E) staining was performed according to standard protocols. Standard sections were made of the hippocampus, cerebellum, and cerebrum in each group of animals.

### Statistical analyses

Values are presented as mean ± SEM. Comparisons among groups were made using analysis of variance (ANOVA) or unpaired Student's t test, as appropriate. P value <0.05 was used as the cutoff for significant findings.

## Results

### SOD activity and quantity

**Figure 1:**
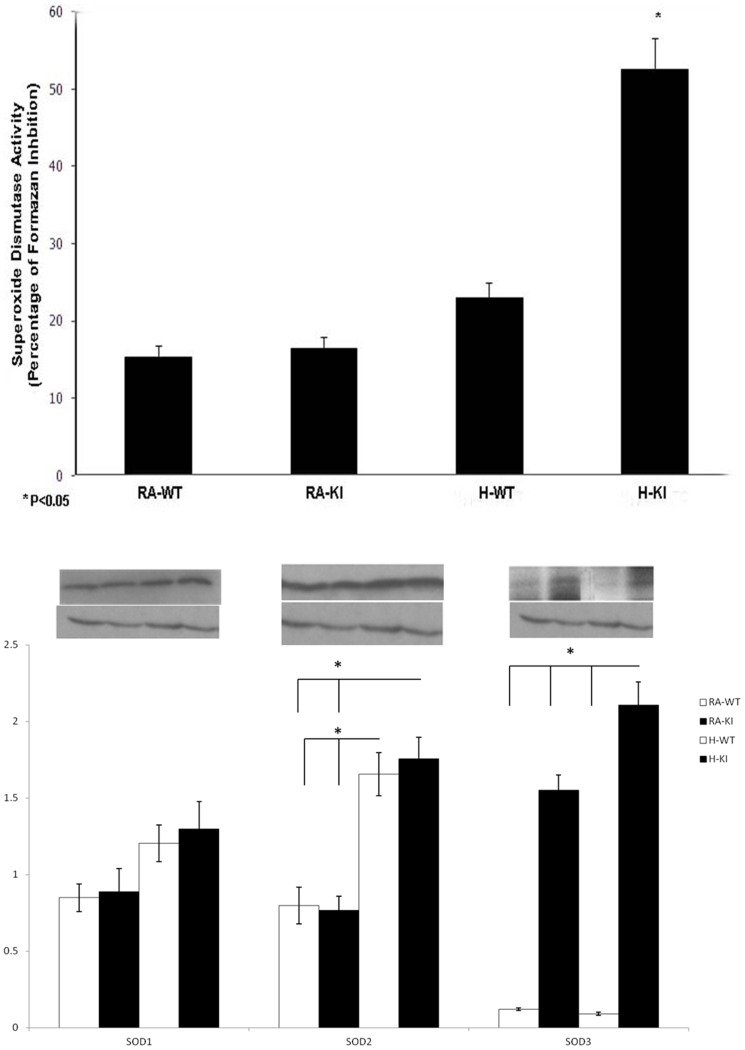
SOD activity ratio in adult mouse brain groups (WT and KI), after exposure to hypoxia (10% for 10 days) in comparison with the RA control groups (WT and KI). Data are mean of 10 animals per group ± SEM. *P<0.05 versus the KI hypoxia and WT hypoxia groups **(Fig. 1A).** Quantitative Western blot for SDO1, SOD2 & hSDO3 (presented as a ratio for B-Actin), in adult mouse brain groups (WT and KI) after exposure to hypoxia (10% for 10 days) in comparison with the RA control groups (WT and KI). Data are mean of 5± SEM animals per group. *P<0.05 HI-KI vs. RA groups and WT hypoxia groups; and H-WT vs. RA groups **(Fig. 1B)**.

Among KI adults, we found SOD activity in brain tissue was statistically significantly higher (x2) than WT adults after exposure to hypoxia (P<0.05) (Figure 1A). To find out which SOD is contributing to the increase in SOD activity, quantitative measure of SOD1, SOD2 and hSOD3 in all brain tissue was done. Our study showed that increased SOD activity after exposure to hypoxia in group KI, is mainly due to increase of both SOD2 and hSOD3 which are increased significantly in KI hypoxic group compared to RA groups and WT hypoxic group (P<0.05) (Fig. 1B). The Beta-actin promoter driving the expression of the human EC-SOD transgene did not lead to augmented expression in KI mice in response to hypoxia, but this significant augmentation in hEC-SOD protein expression in our model could by induced by other stimuli like NO [42].

### Functional studies demonstrate neuronal protection in H-KI mice

**Figure 2:**
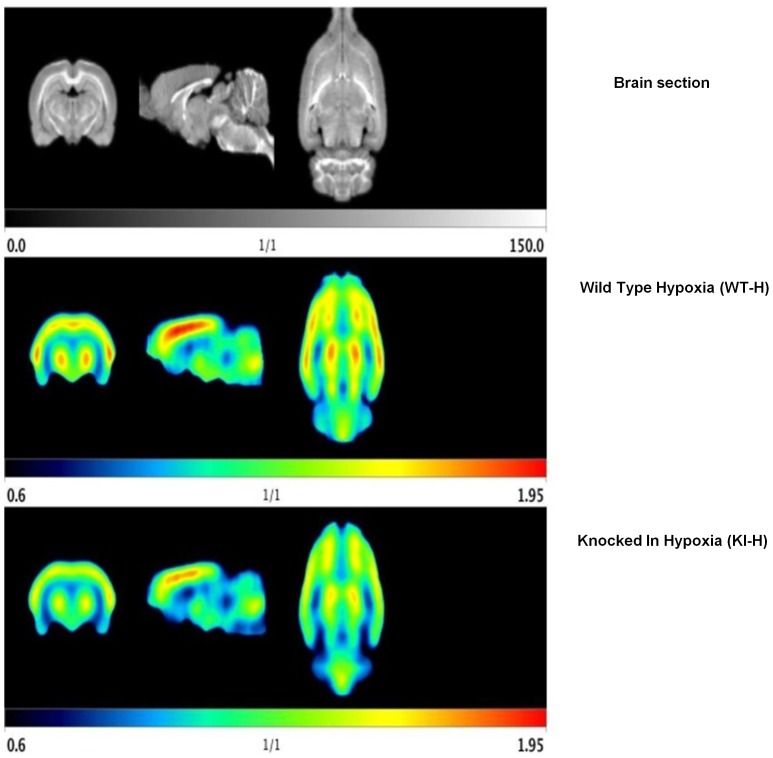
Brain PET scan with an FDG standardized uptake value, showing a cross and longitudinal sections of brain uptake.

PET scans showed that there was a higher uptake of ^18^FDG in hypoxic groups compared to the normoxic groups, and the difference was statistically significant (P<0.05). Interestingly, the ^18^FDG uptake of H-KI brains was lower than H-WT brains in areas sensitive to hypoxia, such as the hippocampus, hypothalamus, thalamus, and medulla (Figure 2 & Table 1), revealing that H-WT brains were maximally activated in their metabolism.

**Table 1:** PET scan data showing FDG in adult mice brain groups (WT and KI) after exposure to hypoxia (10% for 10 days), in comparison with the RA control groups (RA-WT and RA-KI).

Region	RA-WT	RA-KI	H-WT	H-KI	*P value
AcbCore/Shell	0.442	0.436	1.494	0.940	0.003
CaudatePutamen	0.513	0.494	1.727	1.056	0.006
CentralCanal-PAG	0.401	0.396	1.480	0.961	0.004
Pons	0.159	0.156	0.635	0.382	0.004
Septum	0.385	0.372	1.423	0.893	0.001
Medulla	0.220	0.207	0.842	0.490	0.005
Whole Brain	0.349	0.326	1.237	0.754	0.001
Amygdala	0.222	0.209	0.741	0.447	0.002
Cortex-Auditory	0.488	0.450	1.599	0.937	0.002
Cingulate-Ctx	0.607	0.540	2.019	1.223	0.001
Entorhinal-Ctx	0.206	0.195	0.675	0.409	0.003
Frontal-Ctx	0.459	0.355	1.641	0.993	0.002
Insular-Ctx	0.346	0.318	1.218	0.731	0.001
MedialPrefrontal-Ctx	0.558	0.525	1.827	1.130	0.003
Motor-Ctx	0.543	0.447	1.958	1.152	0.001
OrbitoFrontal-Ctx	0.500	0.440	1.802	1.123	0.000
Parietal-Ctx	0.532	0.456	1.809	1.113	0.001
Retrosplenial-Ctx	0.548	0.486	1.871	1.113	0.002
Somatosensory-Ctx	0.539	0.480	1.881	1.132	0.002
Visual-Ctx	0.520	0.471	1.752	1.049	0.001
Hippocampus-Dorsal	0.412	0.381	1.426	0.921	0.003
Hippocampus-Ventral	0.340	0.316	1.277	0.813	0.002
Hypothalamus	0.183	0.177	0.687	0.411	0.001
Olfactory-Ctx	0.248	0.235	0.907	0.581	0.000
pituitary	0.001	0.001	0.005	0.003	0.004
Superior Colliculi	0.519	0.467	1.712	1.061	0.000
Midbrain	0.457	0.441	1.605	0.966	0.003
VTA	0.304	0.310	1.121	0.677	0.005
Cerebellar Grey	0.323	0.321	1.151	0.699	0.008
Cerebellar White	0.392	0.361	1.387	0.850	0.005
InferiorColliculi	0.546	0.497	1.803	1.065	0.001
Thalamus	0.521	0.468	1.776	1.087	0.001

Data are mean ± SEM of 10 animals per group. *P<0.05 versus the KI hypoxia and WT hypoxia groups.

**Figure 3:**
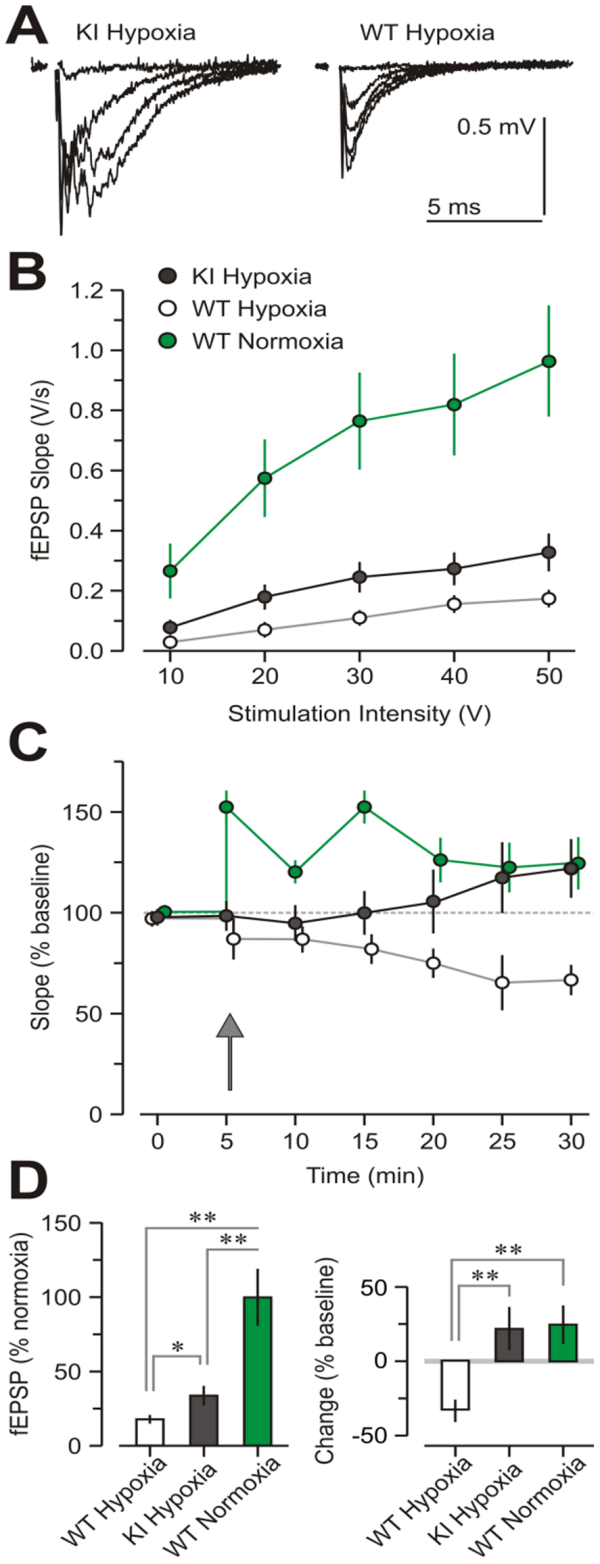
Hippocampal electrophysiological studies showing protected synaptic transmission and plasticity in H-KI slices, when compared to H-WT mice. (A) Representative fEPSPs of CA1 synapses at increasing stimulation strengths. (B) Input-output curves for the different groups. The slope of the fEPSPs in CA1 synapses is plotted against the stimulation intensity to the CA3 afferents. (C) Plot showing the slope of the fEPSPs, with a tetanus applied at 5 min (upward arrow) to induce LTP. (D) Bar graphs showing protection of synaptic transmission (left graph) and plasticity (right graph) in the H-KI group, when compared to the H-WT group. Notably, LTP in the H-KI group is completely normal.

We next evaluated electrophysiological studies in *ex vivo* hippocampal measured with input-output curves for the fEPSPs of CA1 synapses, elicited by stimulation of the CA3 afferents. The data show that synaptic transmission was greatly affected in both hypoxic groups when compared to the RA-WT slices (*P*<0.05). However, the H-KI group showed significantly stronger transmission than the H-WT group (P<0.05), (Figure 3), suggesting that the H-WT synapses were the most affected by hypoxia. Notably, the H-KI group displayed normal long-term synaptic plasticity (LTP), because their LTP measured at 45-min post-tetanic stimulation was similar to the RA-WT group. Conversely, the H-WT slices showed complete absence of LTP (Figure 3).

### Molecular studies showed attenuation of inflammatory and neural cell damage markers in H-KI mice

**Figure 4:**
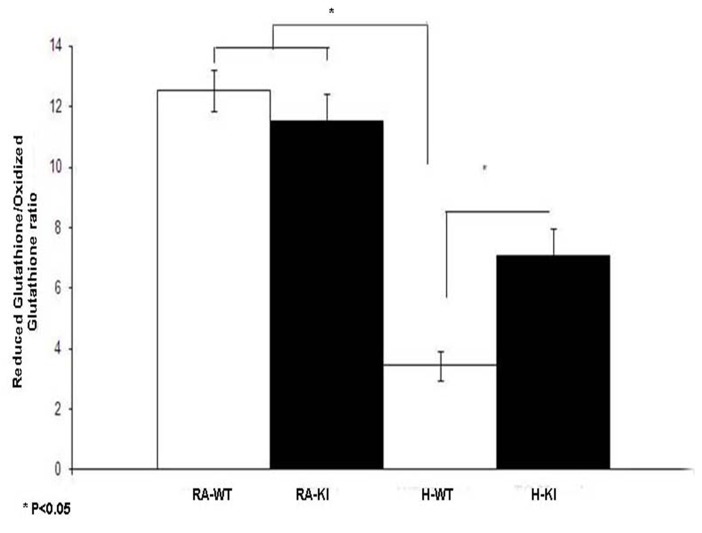
Reduced glutathione/oxidized ratio (GSH / GSSG) in adult mouse brain groups (WT and KI) after exposure to hypoxia (10% for 10 days) in comparison with the RA control groups (WT and KI). Data are mean of 10 animals per group ± SEM. *P<0.05 versus the KI hypoxia and WT hypoxia groups.

GSH/GSSG ratio represents the consumption of reduced glutathione due to accumulation of ROS. GSH/GSSG was significantly lower in the H-WT group compared to the H-KI group (P<0.05). Moreover, there was no difference between the H-KI and RA-KI groups. (Figure 4).

Next, we performed quantitative western blot analysis for inflammatory markers expressed as a ratio of protein density of each marker to Actin protein band density. GFAP expression indicates astrocytes inflammation and damage; it was statistically significantly higher in the H-WT group compared to the H-KI group (P<0.05). There was no difference between the H-KI and RA-KI groups. Iba1 expression, which indicates microglial cells inflammation/damage was statistically significantly higher in the H-WT group compared to the H-KI group (P<0.05). There was no difference between the H-KI and RA-KI groups. MIF expression as an index of systemic inflammation was statistically significantly higher in the H-WT group compared to the H-KI group (P<0.05). There was no difference between the H-KI and RA-KI groups. pAMPK expression as a metabolic marker, was statistically significantly higher in the H-WT group compared to the H-KI group (P<0.05). pACC was also significantly higher in the H-WT group compared to the other three groups, which indicates both increased amounts of pAMPK and pAMPK activity (Table 2).

**Table 2:** Quantitative Assessment of Western blot for molecular markers including: GFAP, IBA1, MIF, pAMPK, and pACC in adult mouse brain groups (WT and KI) after exposure to hypoxia (10% for 10 days), in comparison with the RA control groups (WT and KI).

Markers	RA-WT	RA-KI	H-WT	H-KI
GFAP	0.792±0.04	0.796±0.03	1.426±0.08	0.812±0.08*
Iba1	0.160±0.01	0.162±0.02	0.194±0.01	0.178±0.01*
MIF	0.662±0.07	0.699±0.07	0.935±0.09	0.714±0.02*
pAMPK	0.430±0.12	0.420±0.15	1.378±0.34	0.765±0.19*
pACC	0.276±0.01	0.267±0.01	0.551±0.04	0.369±0.01*

Data are mean of 10± SEM animals per group. *P<0.05 versus the KI hypoxia and WT hypoxia groups.

* P<0.05 (H WT vs. H KI).

### Structural studies showed reduction of cell death and inflammation in H-KI mice

**Figure 5:**
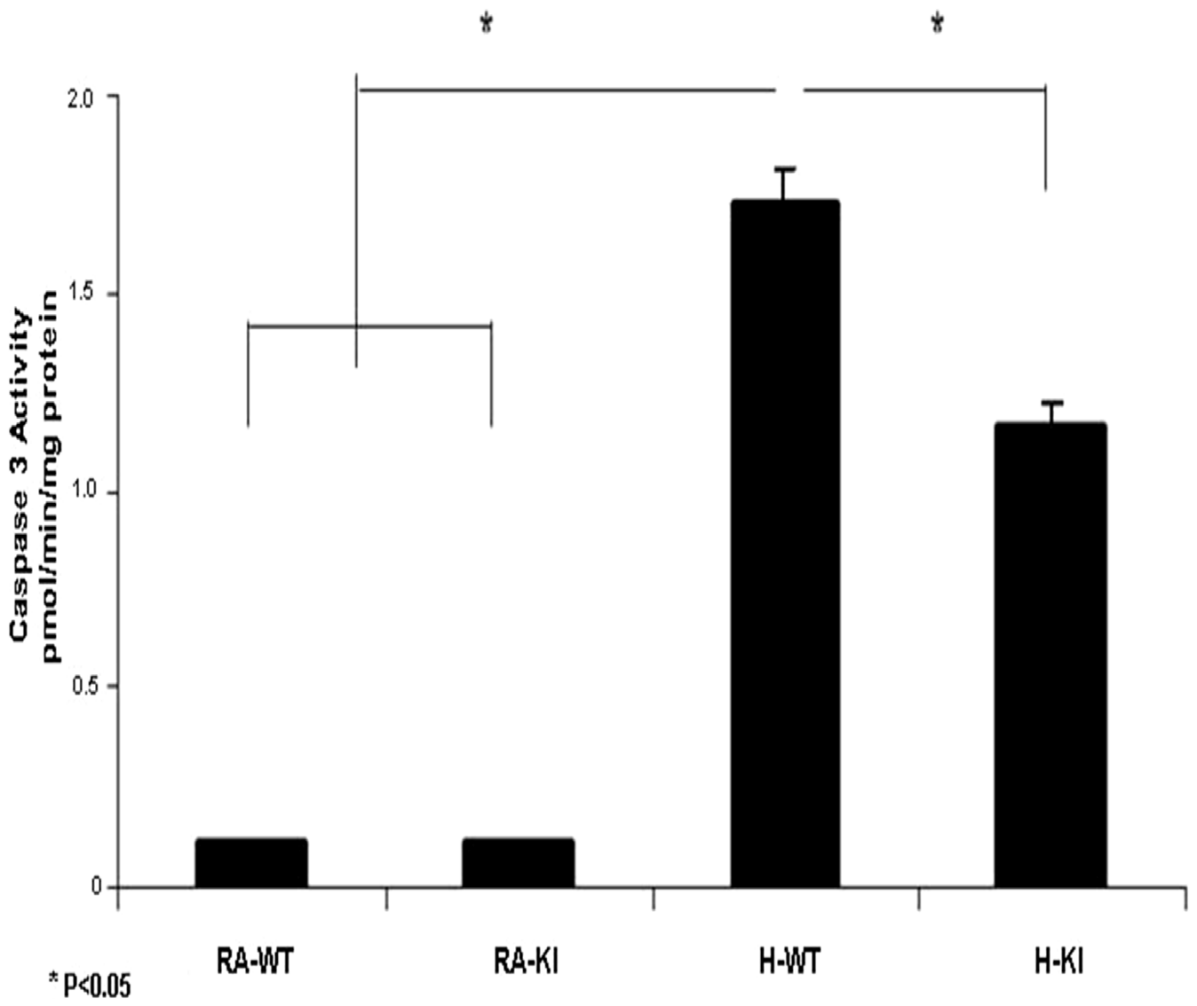
Activated caspase 3 ELISA in adult mouse brain groups (WT and KI), after exposure to hypoxia (10% for 10 days) in comparison with the RA control group (WT and KI). Data are mean of 10 animals per group ± SEM. *P<0.05 versus the KI hypoxia and WT hypoxia groups.

Caspase 3 activity, as a direct evidence of apoptosis, was significantly higher in both hypoxic groups compared to the normoxic groups (P<0.05). However, there was a significantly lower caspase 3 activity in the H-KI group compared to the H-WT group (P<0.05) (Figure 5).

**Figure 6:**
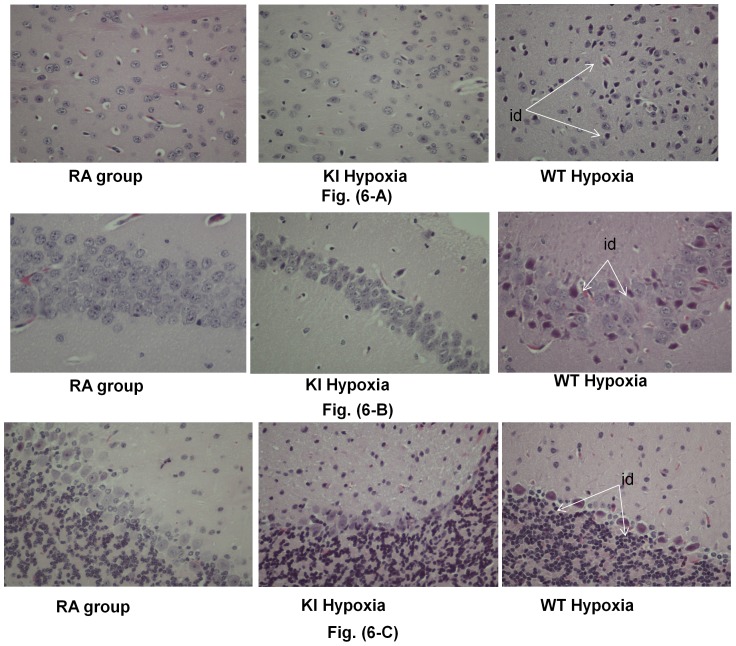
Brain histopathological studies of the control RA and KI hypoxia groups. (A) high-power (40X) H&E staining of cortical brains with normal structure for both the control RA and KI hypoxia groups, while the WT hypoxia group showed injury damage (id). (B) H&E, high-power (60X) view of neurons from the dentate gyrus in the hippocampus with no ischemic damage in both the control and KI hypoxia groups, while the WT hypoxia group showed injury damage (id). (C) H&E, high-power (40X) view of Purkinje cells and granular cells in the cerebellum with no ischemic damage in both the control and KI hypoxia groups, while WT hypoxia group showed injury damage (id).

Sections of the cerebrum, cerebellum or the hippocampus showed no damage (in RA groups) to minimal damage (in H-KI group). However, in the H-WT group, damage was seen in each of the cerebrum, cerebellum, and the hippocampus. Damage was very prominent in the cortical neurons of the grey matter, purkinje neurons in the cerebellum, and the neurons in the dendate gyrus of the hippocampus, which showed ischemic changes (red neuron degeneration) (Figures 6A, B & C).

## Discussion

Adverse impacts of chronic or intermittent hypoxia on development, behavior, and academic achievement have been reported in many well-designed and controlled studies in children, as well as in a variety of studies in adults [43]. Hypoxia, either chronic or intermittent, has been shown to increase oxidative stress and the generation of increased superoxide anion in the brain [44]–[45]. In this study, we showed that overexpression of EC-SOD preserved the excitatory postsynaptic potential and hippocampal neural plasticity after exposure to hypoxia compared to wild type adult mice. We also have shown a clear correlation between overexpression of EC-SOD and decreased brain damage induced by chronic hypoxia exposure as indicated by functional, molecular, and structural studies.

The protection against hypoxia-induced brain damage, offered by overexpression of EC-SOD, could be explained by different mechanisms. The oxidative stress produced by hypoxic insults leads to decreased SOD activity, increasing malondialdehyde (MDA), and lactic acid levels [46]. Inactivation of EC-SOD activity has also been shown to be associated with 100-fold elevation in hypoxia-induced Epo gene expression, compared with wild-type controls. In our KI animal model, there was marked significant increase of SOD activity when compared to WT hypoxia. It is known that human EC-SOD exists as an active and inactive isoform [47]. The marked significant increase of SOD activity in presence of little difference in the amount of EC-SOD among transgenic hypoxic animal vs. wild hypoxic animal, could be explained based on the possibility of having more active EC-SOD in the hypoxic transgenic mice and less inactive EC-SOD and this accounts for the marked change in total SOD activity. When EC-SOD was overexpressed, a significant reduction in Epo gene induction has been shown in hypoxia both *in vitro* and *in vivo* [48]. The inhibitory effect of EC-SOD on hypoxia-induced Epo expression could be due to partial stabilization of HIF-1alpha. It is known that hypoxia generated superoxide radicals are required for induction of HIF-1alpha activity and other downstream target genes [49]–[50]. In KI animals with overexpression of EC-SOD, dismutation of free radicals will be increased compared to the WT group. This leads to decreased levels of ROS including superoxide, which plays a major role in stabilization and activation of HIF-1alpha [51]. The resulted reduction of ROS concentration will decrease HIF-1alpha activation. Modulation of transcription factor HIF-1 alpha and its important gene target VEGF by the antioxidant enzyme EC-SOD was confirmed in other studies [52].

Another important mechanism, which has a great implication in explaining our findings, concerns nitric oxide (NO). NO inhibits hypoxia-inducible transcription of the Epo gene through suppression of HIF-1alpha expression, DNA binding activity, and transcriptional activity [53]–[54]. Under hypoxic conditions, there is a rapid accumulation of superoxide which rapidly reacts with NO to form peroxynitrite and other toxic metabolities [55]–[56]. In KI animals with enhanced expression of EC-SOD, superoxide is dismutated by EC-SOD to form H_2_O_2_. Decreased levels of superoxide would lead to increased physiologic NO concentrations and its bioavailability [57]–[58]. A physiological consequence of this is that EC-SOD influences blood vessel tone by maintaining the biological activity of NO [35].

With overexpression of EC-SOD and dismutation of superoxide, hydrogen peroxide accumulation is important. Many studies have handled this logical assumption by analyzing H_2_O_2_ in different settings. Most of the studies conclude that more SOD does not mean more H_2_O_2_ [59]. The formation of H_2_O_2_ due to dismutation of superoxide, is limited by the amount of superoxide, not by the rate it is converted to H_2_O_2_. Accumulation of superoxide leads to the oxidation of NO with the formation of peroxynitrite. In this situation more H_2_O_2_ is very unlikely to be toxic since this would amount to substituting a very mild cytotoxin (H_2_O_2_) for a very potent one (peroxynitrite). H_2_O_2_ downregulates GRK2 expression which plays a key role in G protein-coupled receptor (GPCR) signaling modulation, and its expression levels are decreased after brain hypoxia/ischemia. Therefore, pharmacological agents effective in the treatment of brain ischaemia/hypoxia should obtain an increase in the level of H_2_O_2_ by blocking GPx, preferably associated to an increased enzymatic activity of SOD and CAT. In the study conducted by [60], either GPx or CAT inhibition enhanced H_2_O_2_ toxicity in rat hippocampal slices, confirming the importance of the integrity of glial antioxidant network [61].

It has been shown that hypoxic exposure (chronic or intermittent) leads to a variety of neurological consequences which can include psychomotor impairment, learning and spatial memory impairment, and in extreme cases, memory retrieval impairment. Reaction time, total number and performance of tasks decrease significantly in hypoxic conditions, and changes in visual sensitivity, attention span, arithmetic and decision making abilities have also been noted [6],[62]–[64]. Imaging studies using PET imaging of H-KI and H-WT mice, showed a marked and significant reduction in FDG uptake in all scanned brain regions including brain regions sensitive to hypoxia mainly the hippocampus, hypothalamus, thalamus, and medulla (Table 1). Increases in ^18^FDG uptake observed in specific brain regions of hypoxic animals, is generally thought to represent an increase demand in glucose metabolism from those intact cells remaining within the affected area. In fact, these alterations suggest a temporal effect of hypoxia on glucose metabolism. Finally, measurements made at times further from the initial insult may demonstrate not only greater, more widespread changes, but also marked decreases in ^18^FDG uptake as a consequence of significant cell loss.

Electrophysiological studies showed that the H-KI group had a significantly stronger transmission than the H-WT group (P<0.05), suggesting that the H-WT synapses were the most affected by hypoxia. Additionally, the H-KI group displayed normal long-term synaptic plasticity, with their LTP, measured at 45-min post-tetanic stimulation, similar to the RA-WT group. Conversely, the H-WT slices showed complete absence of LTP. Our evaluation of the hippocampal CA1-SC circuit does not include any connectivity data, such as synaptic counts, though we believe that this data might add to our understanding of the role of EC-SOD in hippocampal function. This finding could explain the deterioration of both learning and memory in WT mice after exposure to hypoxia and also shows the protective effect of overexpression of EC-SOD in the KI mice group (Fig. 2). We believe that it is the first time that the protective effect of overexpression of EC-SOD has been reported in this setting.

Hypoxic exposure results in a significant increase in pro-inflammatory cytokines including MIF [2]. In neonatal rats with hypoxia-ischemia brain damage, there was a marked increase in the expression of MIF in the brain [64]. In the murine brain, MIF transcripts and protein are mainly present in the cortex, hippocampus, and pituitary gland [65]. In our study there was a significantly marked reduction of MIF expression in brain tissue of H-KI mice compared to the H-WT group (Table 2). This finding supports the anti-inflammatory function of EC-SOD, which could be explained by down regulation of HIF-1alpha and inactivation of NF-KB [55], [66]. In our previous studies, we showed that overexpression of EC-SOD inhibits activation of NF-KB induced by increased ROS in our *in vitro* model [57],[67]. Decreased brain cell inflammation/damage induced by hypoxia was significantly prominent by the significant decrease in the studied inflammatory markers including GFAP, IBA1, and MIF.

In summary, overexpression of EC-SOD and increased its activity has a significant protective effect against chronic hypoxia-induced brain damage. A therapeutic approach that increases EC-SOD protein accumulation, either by overexpression or a therapeutic supplement, could be used prophylactically in patients with long term pathological conditions and chronic disease such as COPD, pulmonary hypertension, sleep disordered breathing and sickle cell disease, all of which are associated with compromised brain oxygenation. A similar approach may also be useful in situations such as hypoxic ischemic insult of newborn, sickle cell disease, stroke or drowning, all of which can lead to impairment of brain function, including learning and spatial memory impairment and psychomotor impairment. Additionally, such a regime could be used as a protective measure for individuals who are operating at the limits of human tolerance and facing relevant operational stressors such as hypoxia, a significant physiological threat at altitude.
